# Immunization status of children at kindergarten entry in Alberta, Canada

**DOI:** 10.17269/s41997-022-00663-3

**Published:** 2022-07-21

**Authors:** Manisha Dhungana, Matthias Hoben, Celine O’Brien, Shannon E. MacDonald

**Affiliations:** 1 Faculty of Nursing, Edmonton Clinic Health Academy, University of Alberta, 11405-87 Ave NW, Edmonton, Alberta T6G 1C9 Canada; 2 Immunization & Communicable Disease Control, Alberta Health, Edmonton, Alberta Canada

**Keywords:** Immunization, Immunization status, Vaccine, Coverage, School entry, Kindergarten, Immunisation, statut vaccinal, vaccin, couverture, entrée à l’école, maternelle

## Abstract

**Objectives:**

Little is known about immunization coverage among kindergarten-aged children in jurisdictions that do not require children’s immunization records to be provided at school entry. Thus, we assessed immunization coverage and associated characteristics of a 2008 birth cohort of Alberta children at kindergarten entry as compared with at the end of grade one.

**Methods:**

This retrospective cohort study used population-based administrative health data for childhood vaccines in Alberta, Canada. We categorized and compared immunization status of children as follows: (a) complete at kindergarten entry; (b) incomplete at kindergarten entry but complete at the end of grade one; and (c) still incomplete at the end of grade one. To assess factors associated with immunization status, we used multinomial logistic regression.

**Results:**

Immunization coverage for the complete vaccine series for children (*N* = 41,515) at kindergarten entry was suboptimal (44.5%, 95% CI 44.0–45.0) and substantially lower than for children at the end of grade one (74.8%, 95% CI 74.3–75.2). Young maternal age, not living with a partner, and having > 1 child in a household were associated with incomplete immunization status at kindergarten entry. Midwife-assisted hospital and home delivery was strongly associated with incomplete immunization status at the end of grade one.

**Conclusion:**

Immunization coverage at kindergarten entry was strikingly low. Risk factors for incomplete immunization status were identified that require particular attention when addressing immunization coverage. The school-based catch-up immunization program in grade one seems to have substantially improved coverage among children, suggesting a potential benefit of shifting the catch-up program from grade one to kindergarten entry.

## Résumé

**Objectifs:**

On en sait peu sur la couverture vaccinale des enfants de la maternelle dans les provinces et territoires qui n’exigent pas la présentation du dossier de vaccination de l’enfant à son entrée à l’école. Nous avons donc évalué la couverture vaccinale et les caractéristiques connexes d’une cohorte d’enfants de l’Alberta nés en 2008 à leur entrée à la maternelle et à la fin de la première année.

**Méthode:**

Cette étude de cohorte rétrospective a fait appel aux données administratives sur la santé de la population de l’Alberta, au Canada, axées sur les vaccins infantiles. Nous avons catégorisé et comparé le statut vaccinal des enfants comme suit : a) complet à l’entrée à la maternelle; b) incomplet à l’entrée à la maternelle, mais complet à la fin de la première année; et c) encore incomplet à la fin de la première année. Pour évaluer les facteurs associés au statut vaccinal, nous avons procédé par régression logistique multinomiale.

**Résultats:**

La couverture vaccinale pour la série vaccinale complète chez les enfants (N = 41 515) à l’entrée à la maternelle était sous-optimale (44,5 %, IC de 95 % 44,0–45,0) et considérablement plus faible que chez les enfants à la fin de la première année (74,8 %, IC de 95 % 74,3–75,2). Le jeune âge maternel, le fait de vivre sans partenaire et le fait d’avoir > 1 enfant dans un ménage étaient associés avec un statut vaccinal incomplet à l’entrée à la maternelle. L’accouchement avec sage-femme à l’hôpital ou à la maison était fortement associé avec un statut vaccinal incomplet à la fin de la première année.

**Conclusion:**

La couverture vaccinale à l’entrée à la maternelle était étonnamment faible. Nous avons cerné des facteurs de risque de statut vaccinal incomplet qui méritent une attention particulière lorsqu’on aborde la couverture vaccinale. Le programme d’immunisation de rattrapage en milieu scolaire durant la première année d’école semble avoir considérablement amélioré la couverture chez les enfants; il pourrait donc être avantageux de l’offrir dès l’entrée à la maternelle plutôt que la première année d’école.

## Introduction

Childhood immunization is one of the most cost-effective interventions to protect children from vaccine-preventable diseases (Government of Alberta, 2017). For many children, kindergarten is often the first time they socialize with a large number of children outside of their household, which can increase their risk of acquiring a vaccine-preventable disease if they are not fully immunized (Busby et al., 2017). Thus, assessing immunization status at kindergarten school entry is critical to determine a child’s risk for vaccine-preventable diseases, as well as the school population’s risk in case of potential outbreaks of disease.

Immunization coverage is defined as the number of people who receive the recommended number of vaccine doses (the numerator) divided by the population eligible to receive the vaccine (the denominator) (MacDonald et al., 2019). Assessing immunization coverage can help to identify groups that are at high risk of vaccine-preventable diseases, as well as potential opportunities for enhancing immunization programs where coverage is lower (Edelstein, 2017; MacDonald et al., 2019). Several studies have investigated factors associated with immunization coverage of children by age 24 and 35 months. Socioeconomic factors such as low family income (Gilbert et al., 2017), low levels of parental education (Gilbert et al., 2017; Niederhauser & Stark, 2005), young maternal age (Bell et al., 2015), unmarried marital status (Bell et al., 2015; Luman et al., 2003), presence of other siblings in a household (Bell et al., 2015; MacDonald et al., 2014), and more than one household move are associated with low immunization coverage (Pearce et al., 2008). Moreover, parents’ knowledge, attitudes, and beliefs regarding immunization, geographic location, birth attendant, and birth location have been highlighted as major factors influencing immunization uptake (Bell et al., 2015; Niederhauser & Stark, 2005). A better understanding of factors that influence immunization coverage at school entry could help policymakers to establish strategies to increase coverage.

In Canada, vaccine programs and schedules vary by jurisdiction (Busby et al., 2017). Only three of thirteen provinces and territories require parents to show proof of their child’s immunization status to attend school (Adedzi & Dube, 2020; Busby et al., 2017; Lee & Robinson, 2016). Data collection and availability on vaccine events also varies between provinces/territories and thus cross-provincial comparisons are challenging (Busby et al., 2017). In the province of Alberta, routine childhood immunizations are only administered by public health nurses (PHNs) during appointments at public health clinics (Busby et al., 2017); no routine immunizations for this age group are provided by physicians or pharmacists, which ensures that public health records are complete. Immunization coverage for most routine childhood vaccines is measured and reported at two and seven years of age (the latter being approximately at the end of grade one), but is not assessed at school entry (i.e., at the start of kindergarten when most children are five years of age) (Busby et al., 2017; Government of Alberta, 2017). During the grade one school year, PHNs review all student records and undertake efforts to catch-up children on missed vaccines. While numerous studies have assessed risk factors of incomplete immunization at two years of age (Bell et al., 2015; MacDonald et al., 2014; Nestander et al., 2018), risk factors of incomplete immunization at kindergarten entry are not as well investigated. Therefore, our aim was (1) to estimate the change in immunization coverage in a province-wide cohort of children in Alberta at the start of kindergarten and the end of grade one, and (2) to identify risk factors associated with incomplete immunization status at the start of kindergarten.

## Methods

### Study setting and population

This retrospective cohort study used population-based administrative data in Alberta, a Canadian province with a population of approximately 4.5 million (Government of Alberta, 2022). We assessed the immunization coverage of a province-wide cohort of children born in Alberta in 2008 when they were at the start of kindergarten and end of grade one, including children who received their education through homeschooling (typically < 2% of the population of Albertan schoolchildren are homeschooled in a given school year) (Van Pelt, 2015). All routine childhood immunizations in Alberta are covered by a universal provincially funded health care insurance plan (Bell et al., 2015). The recommended vaccines for preschool children during the study period were diphtheria, pertussis, tetanus, polio, *Haemophilus influenzae* type b (DTaP-IPV-Hib); meningococcal conjugate (Men C); pneumococcal conjugate (PCV); and measles, mumps, rubella (MMR), or MMR-varicella (MMRV) (see Table 1). We excluded (1) children with health care cancellation by age 7 (death or migration from the province), (2) First Nations children living on reserves, who receive vaccines through federal programs, and (3) children living in the town of Lloydminster (whose vaccines are administered by the neighbouring province). We included only one child of any multiple births (e.g., twins), selected randomly, to ensure the independence of observations.

**Table 1:** Alberta recommended routine preschool childhood immunization schedule that applies to a 2008 birth cohort

Vaccines	2 months	4 months	6 months	12 months	18 months	4–6 years^b^	Total doses
Diphtheria, tetanus, acellular pertussis, polio, *Haemophilus influenzae* type b (DTaP-IPV-Hib)	X	X	X		X	X (does not contain Hib)	5
Pneumococcal conjugate (PCV)	X	X	X		X		4
Meningococcal conjugate (Men C)	X	X		X			3
Measles-containing vaccine, administered as either measles, mumps, rubella (MMR), or MMR-varicella (MMRV)^a^				X		X	2
Varicella-containing vaccine, administered either as varicella (alone) or MMRV^a^				X		(X)	1 or 2
Complete vaccine schedule							14–15

^a^This schedule reflects changes to the vaccine schedule that occurred in 2012 that applies to the 2008 birth cohort. Specifically, the 2^nd^ dose of varicella was added for 4- to 6-year-old children in Aug 1, 2012. Therefore, children who turned 4 years old before Aug 1, 2012, would not be eligible to receive a second dose of varicella vaccine, and thus would have received MMR vaccine instead of MMRV

^b^Although these doses are offered to children at 4–6 years of age, it is recommended that they receive them before they begin school, in order to ensure that they are fully protected

### Data sources

Three administrative data sources were linked to form this cohort: Vital Statistics, Alberta Health Care Insurance Plan Central Stakeholder Registry (AHCIP/CSR), and Immunization and Adverse Reaction to Immunization (Imm/ARI). Vital Statistics includes information on any infant born in Alberta, as well as infant and maternal characteristics at time of birth, and was used to identify the study population. The AHCIP/CSR includes demographic information of all residents of Alberta, including First Nations status, death, and people who migrated from Alberta to another province. Almost all Albertans (99%) are registered with AHCIP/CSR and are issued a unique lifetime identifier (ULI), which enables linkage of administrative databases. To determine immunization status, the cohort was linked to the Imm/ARI database, which records immunization information for all provincially funded vaccines administered in Alberta, and has guidelines and business rules for data submission, thus increasing data integrity, quality, and completeness (Bell et al., 2015).

### Outcome variables and potential associated factors

**Fig. 1:**
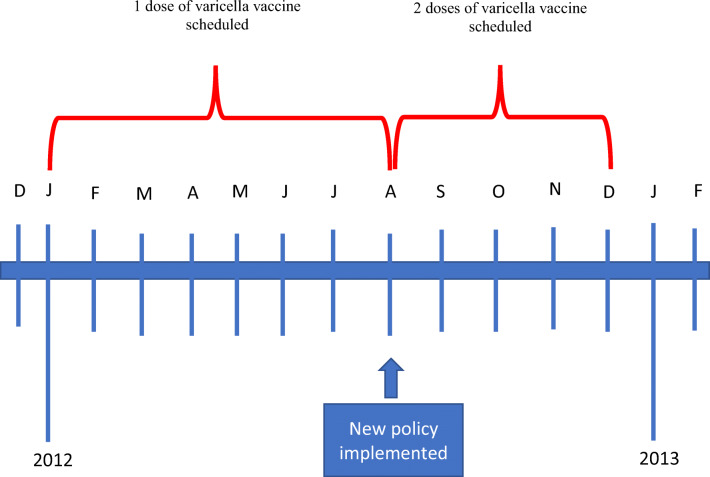
Timeline of the change in Alberta’s varicella immunization program in 2012

We had two outcomes of interest:

1. *Immunization coverage for each individual vaccine* (which included DTaP-containing vaccines, PCV, Men C, MMR/MMRV, and varicella) at the start of kindergarten and at the end of grade one. *Immunization coverage* was defined as the number of children who had received the recommended number of vaccine doses, divided by the eligible population. Vaccine coverage for each individual vaccine series was categorized as *complete* (all doses in the vaccine series) or *incomplete* (some or no doses). Only valid doses were included in coverage assessments, as doses administered earlier than the minimum age and/or with less than the minimum interval can lead to a decreased level of protection. We determined the validity of each vaccine dose according to provincial recommendations for minimum age of receipt and minimum intervals between vaccine doses (Alberta Health Services, 2013). Invalid doses (which constituted < 0.5% of all doses administered in our study) were not included in the determination of immunization status. Due to a change in the varicella vaccine schedule partway through the study period (see Fig. 1), children born between January 1, 2008 and August 1, 2008, were considered *complete* if they received one dose of varicella vaccine, while children born after August 1, 2008, needed two doses of varicella vaccine.
2. *Immunization coverage for the entire vaccine schedule* (used as the outcome variable in our multinomial model) included all scheduled doses for children at the start of kindergarten and at the end of grade one (see Table 1). *Complete vaccination* was defined as receiving all vaccine doses (i.e., 14 or 15 doses, dependent on age) by the scheduled age. *Incomplete vaccination* was defined as receiving only some doses or no doses of the vaccines at the scheduled age. Furthermore, we categorized immunization status of children for the entire vaccine schedule into three categories: (a) complete at kindergarten entry; (b) incomplete at kindergarten entry, but complete at the end of grade one; or (c) still incomplete at the end of grade one.

We examined the relationship between immunization status and potential associated factors, including maternal age, marital status (i.e., living with a partner or not), household income quintile, zone of residence, child sex, type of birth attendant and location, gestational term, number of children, and number of household moves. Number of household moves by age 7 was obtained from AHCIP/CSR. All other variables were extracted from the Vital Statistics database and were measured at the time of birth. Father’s demographics were not included as they were highly correlated with the mother’s demographics.

### Data analysis

We calculated proportions and 95% confidence intervals (CIs) for immunization coverage for each vaccine separately and combined on September 1, 2013 (start of kindergarten), and again on June 30, 2015 (end of grade one). To assess the association between each factor and immunization status, we performed a multinomial logistic regression. Our outcome variable (immunization status) had three categories: complete at kindergarten entry (reference category); incomplete at kindergarten entry, but complete at the end of grade one (comparison group 1); and still incomplete at the end of grade one (comparison group 2). We reported adjusted odds ratios (aOR) and 95% CIs. Potential associated factors were included in the model based on evidence from previous research and were retained in the model regardless of statistical significance. Multicollinearity between all variables was confirmed, with all variance inflation factors measuring lower than 2. Model fit was assessed using the chi-square goodness-of-fit test and pseudo *R*^2^.

## Results

After exclusions (*n* = 8634), the final cohort included 41,515 children. Table 2 presents the demographic characteristics of the cohort by their immunization status. Immunization coverage for most individual vaccines and for all vaccines combined was low at the start of kindergarten, increasing by the end of grade one (see Table 3). DTaP-containing vaccine coverage was the lowest at kindergarten entry at 47.5% (95% CI 47.0–48.0), followed by MMR/MMRV at 49.1% (95% CI 48.6–49.5). By the end of grade one, coverage for DTaP-containing vaccine was 81.7% (95% CI 81.4–82.1) and MMR/MMRV coverage was 82.8% (95% CI 82.5–83.2). PCV vaccine coverage was only slightly lower at kindergarten entry (84.1%, 95% CI 83.8–84.5) than at the end of grade one (85.2%, 95% CI 84.8–85.5) and Men C coverage did not significantly improve between kindergarten entry and grade one. For all vaccines combined, children at kindergarten entry had 30.3% (95% CI 44.0–75.2) lower immunization coverage compared to the end of grade one.

**Table 2:** Characteristics of children according to their immunization status

Variables	Category	Complete at K entry (*N* = 18,478) *n* (%)	Incomplete at K entry, but complete at the end of G1 (*N* = 12,563) *n* (%)	Still incomplete at the end of G1 (*N* = 10,474) *n* (%)
Maternal age	< 21	1203 (6.5)	932 (7.4)	1199 (11.4)
	21–25	3029 (16.4)	2030 (16.2)	2126 (20.3)
	26–30	6536 (35.4)	4311 (34.3)	3410 (32.6)
	31–35	5333 (28.9)	3663 (29.2)	2597 (24.8)
	36–40	2108 (11.4)	1453 (11.6)	957 (9.1)
	> 40	269 (1.5)	174 (1.4)	185 (1.8)
Maternal marital status	Living with a partner	14,619 (79.1)	9389 (74.8)	7133 (68.3)
	Without a partner	3851 (20.9)	3163 (25.2)	3318 (31.7)
Household income quintile	Q5 (highest)	4251 (23.0)	3009 (24.0)	2091 (20.0)
	Q4	3948 (21.4)	2681 (21.3)	2114 (20.2)
	Q3	3234 (17.5)	2303 (18.3)	1838 (17.5)
	Q2	3480 (18.8)	2306 (18.4)	2092 (20.0)
	Q1 (lowest)	3565 (19.3)	2264 (18.0)	2338 (22.3)
Zone of residence	Calgary	7837 (42.4)	4724 (37.6)	3574 (34.1)
	Edmonton	5505 (29.8)	4131 (32.9)	2848 (27.2)
	North	2073 (11.2)	1168 (9.3)	1753 (16.7)
	South	1683 (9.1)	801 (6.4)	852 (8.1)
	Central	1377 (7.5)	1735 (13.8)	1444 (13.8)
Sex of child	Male	9464 (51.2)	6521 (51.9)	5384 (51.4)
	Female	9014 (48.8)	6042 (48.1)	5090 (48.6)
Type of birth attendant and location	Physician at hospital/other & other at hospital/en route	18,316 (99.1)	12,418 (98.8)	10,045 (95.9)
	Midwife at hospital/en route	105 (0.6)	85 (0.7)	167 (1.6)
	Midwife at home/other	34 (0.2)	37 (0.3)	204 (1.9)
	Other at other	23 (0.1)	23 (0.2)	58 (0.6)
Gestational term	Term or more	17,146 (92.8)	11,678 (93.0)	9708 (92.7)
	Pre-term	1332 (7.2)	885 (7.0)	766 (7.3)
Number of children in a household (including index child)	1	8968 (48.5)	5252 (41.8)	4050 (38.7)
	2	6426 (34.8)	4666 (37.1)	3370 (32.2)
	3	2151 (11.6)	1799 (14.3)	1707 (16.3)
	≥ 4	933 (5.0)	846 (6.7)	1347 (12.9)
Household moves	≤ 1	15,524 (84.0)	10,427 (83.0)	8333 (79.6)
	≥ 2	2954 (16.0)	2136 (17.0)	2141 (20.4)

*Abbreviations. G1* grade 1, *K* kindergarten

**Table 3:** Immunization coverage of the 2008 cohort of Alberta children at the start of kindergarten and at the end of grade one (*N* = 41,515)

Vaccine type	Coverage			
	Kindergarten		Grade 1	
	*n*	% (95% CI)	*n*	% (95% CI)
DTaP-containing	19,724	47.5% (47.0–48.0)	33,933	81.7% (81.4–82.1)
Measles-containing (MMR/MMRV)	20,370	49.1% (48.6–49.5)	34,383	82.8% (82.5–83.2)
Men C	36,106	87.0% (86.6–87.3)	36,124	87.0% (86.7–87.3)
PCV	34,932	84.1% (83.8–84.5)	35,361	85.2% (84.8–85.5)
Varicella-containing (varicella/MMRV)	28,163	67.8% (67.4–68.3)	36,050	86.8% (86.5–87.2)
All vaccines	18,478	44.5% (44.0–45.0)	31,041	74.8% (74.3–75.2)

*Abbreviations. DTaP* diphtheria, tetanus, acellular pertussis, *G1* grade 1, *K* kindergarten, *Men C* meningococcal conjugate, *MMR/MMRV* measles, mumps, rubella, varicella, *PCV* pneumococcal conjugate

Table 4 shows the factors associated with children’s immunization status in the multinomial logistic regression model. Compared to children who were completely immunized at kindergarten entry, the likelihood of being incompletely immunized at kindergarten entry, but completely immunized at the end of grade one and still incompletely immunized at the end of grade one was greater in children whose mothers were younger or not living with a partner. Additionally, children from low-income households (Q1 and Q2) were 0.83 (95% CI 0.77–0.89) and 0.87 (95% CI 0.81–0.94) times less likely to be incompletely immunized at kindergarten entry, but completely immunized at the end of grade one, compared to children from families with the highest household income (Q5). In contrast, the odds of still being incompletely immunized at the end of grade one was higher in children from families with the lowest household income (Q1) compared to children from high-income families (aOR = 1.15, 95% CI 1.07–1.25). Compared to Calgary zone children, those from Edmonton and Central zones were more likely to be incompletely immunized at kindergarten entry, but completely immunized at the end of grade one, with aORs of 1.23 (95% CI 1.17–1.30) and 2.00 (95% CI 1.85–2.17), respectively, whereas those from North and South zones were less likely to be incompletely immunized at kindergarten entry, but completely immunized at grade one with aORs of 0.86 (95% CI 0.79–0.94) and 0.77 (95% CI 0.70–0.84), respectively. The odds of still being incompletely immunized at the end of grade one was lower in children who were born in the South zone (aOR = 0.89, 95% CI 0.81–0.98), and higher in those who were born in North (aOR = 1.55, 95% CI: 1.43–1.68) and Central (aOR = 1.94, 95% CI: 1.77–2.11) zones, compared to Calgary zone.

**Table 4:** Multinomial logistic regression of factors associated with immunization status

Complete at K entry (ref)	Incomplete at K entry, but complete at the end of G1 aOR (95% CI)	*P*-value	Still incomplete at the end of G1 aOR (95% CI)	*P*-value
Maternal age (ref = > 40)				
< 21	1.28 (1.03–1.59)	0.03	1.79 (1.44–2.23)	< 0.001
21–25	1.13 (0.92–1.38)	0.25	1.31 (1.06–1.61)	0.01
26–30	1.12 (0.92–1.36)	0.28	0.99 (0.81–1.22)	0.95
31–35	1.14 (0.93–1.38)	0.21	0.88 (0.72–1.08)	0.23
36–40	1.13 (0.92–1.38)	0.26	0.77 (0.62–0.95)	0.02
Maternal marital status (ref = living with partner)				
Without partner	1.31 (1.24–1.40)	< 0.001	1.55 (1.45–1.65)	< 0.001
Household income quintile (ref = Q5)				
Q4	0.90 (0.84–0.97)	0.04	1.09 (1.01–1.18)	0.03
Q3	0.93 (0.87–1.00)	0.07	1.07 (0.98–1.16)	0.11
Q2	0.87 (0.81–0.94)	< 0.001	1.12 (1.03–1.21)	0.006
Q1 (lowest)	0.83 (0.77–0.89)	< 0.001	1.15 (1.07–1.25)	< 0.001
Zone of residence (ref = Calgary)				
Edmonton	1.23 (1.17–1.30)	< 0.001	1.05 (0.99–1.12)	0.12
North	0.86 (0.79–0.94)	< 0.001	1.55 (1.43–1.68)	< 0.001
South	0.77 (0.70–0.84)	< 0.001	0.89 (0.81–0.98)	0.01
Central	2.00 (1.85–2.17)	< 0.001	1.94 (1.77–2.11)	< 0.001
Sex of child (ref = male)				
Female	0.97 (0.93–1.02)	0.20	0.99 (0.94–1.04)	0.67
Type of birth attendant and location (ref = physician/other at hospital & other/hospital & en route)				
Midwife at hospital/en route	1.18 (0.88–1.58)	0.26	3.71 (2.89–4.77)	< 0.001
Midwife at home/other	1.59 (0.99–2.53)	0.05	13.54 (9.36–19.58)	< 0.001
Other at other	1.41 (0.78–2.55)	0.26	4.04 (2.44–6.68)	< 0.001
Gestational term (ref = term or more)				
Pre-term	0.97 (0.89–1.06)	0.48	1.03 (0.94–1.13)	0.54
Number of children in a household (including index child) (ref = 1)				
2	1.30 (1.23–1.36)	< 0.001	1.37 (1.29–1.45)	< 0.001
3	1.50 (1.40–1.62)	< 0.001	2.15 (1.99–2.32)	< 0.001
≥ 4	1.71 (1.54–1.90)	< 0.001	4.05 (3.67–4.47)	< 0.001
Number of moves (ref = ≤ 1)				
≥ 2	1.04 (0.97–1.11)	0.27	1.12 (1.05–1.20)	< 0.001

*Abbreviations*. *aOR* adjusted odds ratio, *CI* confidence interval, *G1* grade 1, *K* kindergarten

Compared to physician delivery at a hospital, those with a midwife delivery at a hospital had 3.71 times (95% CI 2.89–4.77) higher odds of still being incompletely immunized at the end of grade one rather than completely immunized at kindergarten entry. The odds of incomplete immunization at the end of grade one was even higher for midwife delivery at home (aOR = 13.54, 95% CI 9.36–19.58). There was a stepwise increase in the odds of being incompletely immunized at the end of grade one with the number of children in a household. Children in households with two (aORs = 1.30 [95% CI 1.23–1.36]), three (1.50 [95% CI 1.40–1.62]), or four or more children (1.71 [95% CI 1.54–1.90]) had higher odds of being incompletely immunized at kindergarten entry, but completely immunized at the end of grade one, compared to children in households with one child; i.e., children in larger households were less likely to be completely immunized. Similarly, the odds of being still incompletely immunized at the end of grade one was 1.12 (95% CI 1.05–1.20) times greater in children who had moved residence two times or more, compared to those who had moved one time or less. Incomplete immunization at kindergarten entry or the end of grade one was not associated with child sex or gestational term.

## Discussion

The low levels of complete immunization status of children upon entry to kindergarten in our cohort were concerning but increased by 30% for all vaccines combined after a grade one catch-up program. Factors associated with incomplete immunization included young maternal age, mothers not living with a partner, and having two or more children in a household.

### Immunization coverage for each vaccine and for all vaccines combined

Immunization coverage for most individual vaccines and all vaccines combined was strikingly low at the start of kindergarten. The lowest coverage at kindergarten entry was for vaccines with doses due at 4–6 years of age (DTaP-containing vaccine [47.5%], MMR/MMRV [49.1%]), versus vaccines due at 18 months (PCV [84.1%]) or 12 months (Men C [87.0%]). This finding of decreased coverage with increasing age of scheduled dose is consistent with previous findings (Hermann et al., 2019). A possible explanation for this phenomenon could be that mothers in Canada often return to work after a subsidized maternity leave after 1 year (Bell et al., 2015; Saini et al., 2017). The double duty of parenthood and paid work or needing to obtain time off from work may present logistical challenges to accessing immunizations (Hill et al., 2015; Saini et al., 2017). As such, making immunization appointments more easily available through longer clinic hours or drop-in services may help to address these logistical challenges.

These findings are important as low immunization coverage leaves children vulnerable to vaccine-preventable diseases. There have been multiple vaccine-preventable disease outbreaks in communities or regions in Alberta with substantial numbers of unimmunized children, such as a measles outbreak in Southern Alberta in 2013 (Kershaw et al., 2014) and an outbreak of pertussis in the same region in 2019 (Alberta Health Services, 2020).

Vaccine coverage in our cohort increased substantially at the end of grade one, likely due to the grade one catch-up immunization program. In this program, PHNs review immunization records of students in grade one, identify missing immunizations, send home immunization consent forms, and administer immunizations at school. Any child who has an incomplete immunization record on file is contacted up to three times (Alberta Health Services, n.d.). The exception to this notable increase at the end of grade one was Men C and PCV vaccines, which already had relatively high coverage at kindergarten entry, likely because the final required dose of these vaccines is scheduled at 12 or 18 months of age, whereas the other vaccines required an additional dose at 4–6 years. Our study findings are consistent with previous studies (Adedzi & Dube, 2020; Rehn et al., 2016; Riddell et al., 2001) that found school-based catch-up immunization programs to be effective in improving immunization uptake. Therefore, moving this catch-up program to an earlier time point, either in kindergarten or prior to kindergarten entry, may maximize the number of fully vaccinated children early on, thereby reducing the amount of time children are susceptible to contracting vaccine-preventable diseases. In addition, to address the possibility that parents may be uncomfortable with their kindergarten-aged child receiving vaccines without their presence, PHNs could assess children’s immunization status at kindergarten entry and invite parents to bring their child to a public health centre or be immunized at school.

Even though vaccine coverage at the end of grade one increased compared to kindergarten entry, there were still some children who remained incompletely immunized at the end of grade one. Children being incompletely immunized even after being exposed to the catch-up program may be due to parental vaccine hesitancy or refusal to immunize their children, as opposed to logistical challenges, as highlighted above. To address parental vaccine hesitancy, one possible strategy might be to have PHNs build a trusting relationship with these parents by personally reaching out and engaging with them in discussion about childhood immunizations, providing relevant evidence to address their queries/concerns, and eliminating any misconceptions (Gust et al., 2008; McKee & Bohannon, 2016).

### Factors associated with children’s incomplete immunization status

Previous studies have assessed risk factors for incomplete immunization of children at 2–3 years old (Bell et al., 2015; Luman et al., 2003; MacDonald et al., 2014; Nestander et al., 2018). Our study adds to this literature by identifying risk factors at kindergarten entry. Having a mother who was younger and/or not living with a partner increased a child’s risk for incomplete immunization, as seen with previous studies of younger children (Bell et al., 2015; Luman et al., 2003).

Children from the lowest income households were more likely to be completely immunized at the start of kindergarten. Although we were surprised by this finding, there have been contradictory findings on this issue, with some previous Canadian studies (Bell et al., 2015; Gilbert et al., 2017) finding that low household income is associated with incomplete immunization status, whereas another study has found no association (Wilson et al., 2018). Despite the history of outbreaks and low childhood immunization coverage in specific areas of Southern Alberta (Alberta Health Services, 2020; Kulig et al., 2002; Matkin et al., 2014), we found that children from the South zone overall were more likely to be completely immunized at the start of kindergarten.

Midwife-assisted home and hospital delivery were significantly associated with incomplete immunization status of children at the end of grade one. In previous studies, births attended by a midwife were linked with non-immunization status of children at age two (Bell et al., 2015). We are unable to conclude from our study whether the mother’s decision was influenced by the midwife’s attitude and beliefs or if mothers who are likely to refuse vaccines seek out midwife-attended births (Bell et al., 2015; Sahni et al., 2014). The mandate of the midwifery profession in Canada is to stay neutral and not give advice on immunization, while leaving the decision up to parents (Dube et al., 2013; Dube et al., 2016). Further research is needed in order to fully understand the relationship between midwife delivery and immunization decisions.

Also, consistent with previous studies (Bell et al., 2015; MacDonald et al., 2014; Niederhauser et al. 2005), we found that having more than one child in a household and a greater number of household moves were also associated with incomplete immunization status at kindergarten entry. The presence of more than one of these factors may indicate a stressful life where parents (particularly mothers) have to manage multiple competing priorities, making it challenging to schedule and attend immunization appointments (Bell et al., 2015). While these logistical challenges to immunization are not easily modifiable, understanding what puts individuals and populations at risk is key to monitoring these vulnerable populations, and developing and testing interventions tailored to these groups, such as providing additional reminders and immunization outreach services (Bell et al., 2015; Cushon et al., 2012; Williams et al., 2011).

### Strengths and limitations

We assessed immunization status of a cohort of children at two time points: at kindergarten entry and at the end of grade one after a catch-up immunization program was offered. We used population-based, administrative health data from a province with a single immunization delivery and record-keeping system, which ensured that our dataset contained complete childhood immunization records.

There were some limitations of this study. We assessed immunization coverage of children on September 1, 2013, assuming that most of the children had started kindergarten at this point. However, due to the cut-off point for kindergarten entry during the study period (March 1), children born in January and February 2008 may have entered kindergarten in the prior year and be enrolled in grade one on September 1, 2013, and thus might have already been caught up on their vaccines by PHNs. Therefore, it is possible that this study may have overestimated coverage at kindergarten entry.

The dataset used for this study did not contain information regarding the history of varicella disease, which may have made varicella immunization unnecessary. Therefore, some children who were classified as incomplete for varicella vaccine might not actually need the vaccine. However, this was likely a small number of children, given that varicella disease was no longer circulating in the community due to the success of the immunization program (Waye et al., 2013).

Furthermore, there may be residual confounding due to unmeasured variables that could have influenced immunization coverage. Additional potential associated factors, such as maternal education, geographical location, and residence (Luman et al., 2003; Niederhauser et al. 2005), would have been useful to examine but were not available in the dataset. Finally, as this study focused on children in Alberta, where early childhood immunizations are delivered solely by PHNs at public health clinics, results may not be generalizable to jurisdictions that use other types of delivery, such as physician delivery.

## Conclusion

This study suggests that immunization coverage at kindergarten entry is far below the levels required to prevent spread of vaccine-preventable diseases, but that the school-based catch-up immunization program in grade one substantially increased coverage. Thus, it is likely that immunization policies and programs that provide school-based catch-up immunizations for children at the start of kindergarten would be highly beneficial. This study also contributes new evidence on factors associated with a child having an incomplete immunization status at the start of kindergarten. Public health approaches such as extending immunization clinic hours, providing drop-in services, sending immunization appointment reminders, providing immunization outreach services, and establishing a trusting relationship with parents have the potential to improve immunization coverage.

## Contributions to knowledge

What does this study add to existing knowledge?

- In the absence of a school entry requirement, vaccine coverage is strikingly low among kindergarten-aged children.
- The reasons children are incompletely immunized on school entry differ from the reasons they are incompletely immunized after a vaccine catch-up program.

What are the key implications for public health interventions, practice, or policy?

- Catch-up immunization programs are effective at increasing coverage, but should be implemented before entry to school.
- Public health approaches such as extending immunization clinic hours, providing drop-in services, sending immunization appointment reminders, providing immunization outreach services, and establishing a trusting relationship with parents have the potential to improve immunization coverage.

## Data Availability

The data steward is the Alberta Ministry of Health.

## References

[CR1] Adedzi, K. A., Dube, E. (2020). Overview of Canadian school-based immunization programs. https://canvax.ca/brief/overview-canadian-school-based-immunization-programs. Accessed March 9, 2022.

[CR2] Alberta Health Services. (2013). Standard for recommended immunization schedules. 2013. https://www.albertahealthservices.ca/assets/info/hp/cdc/if-hp-cdc-ipsm-recommended-immunization-schedule-03-110.pdf. Accessed August 10, 2020.

[CR3] Alberta Health Services. (2020). Pertussis outbreak declared over in AHS South zone. https://www.albertahealthservices.ca/news/releases/2020/Page15398.aspx#:~:text=LETHBRIDGE%20%E2%80%93%20An%20outbreak%20of%20pertussis,with%20no%20hospitalizations%20or%20deaths. Accessed March 13, 2022.

[CR4] Alberta Health Services. (n.d.). Immunization-school services. https://www.albertahealthservices.ca/findhealth/service.aspx?Id=4209. Accessed February 10, 2020.

[CR5] Bell CA, Simmonds KA, MacDonald SE (2015). Exploring the heterogeneity among partially vaccinated children in a population-based cohort. Vaccine.

[CR6] Busby, C., Jacobs, A., Muthukumaran, R. (2017). In need of a booster: How to improve childhood vaccination coverage in Canada. *Commentary No. 477*. https://ideas.repec.org/a/cdh/commen/477.html. Accessed May 10, 2018.

[CR7] Cushon JA, Neudorf CO, Kershaw TM, Dunlop TG, Muhajarine N (2012). Coverage for the entire population: Tackling immunization rates and disparities in Saskatoon health region. Canadian Journal of Public Health.

[CR8] Dube E, Vivion M, Sauvageau C, Gagneur A, Gagnon R, Guay M (2013). How do midwives and physician discuss childhood vaccination with parents?. Journal of Clinical Medicine.

[CR9] Dube E, Vivion M, Sauvageau C, Gagneur A, Gagnon R, Guay M (2016). “Nature does things well, why should we interfere?”: Vaccine hesitancy among mothers. Qualitative Health Research.

[CR10] Edelstein M (2017). Measuring vaccination coverage better will help achieve disease control. International Health.

[CR11] Gilbert NL, Gilmour H, Wilson SE, Cantin L (2017). Determinants of non-vaccination and incomplete vaccination in Canadian toddlers. Human Vaccines & Immunotherapeutics.

[CR12] Government of Alberta. (2017). Alberta immunization strategy 2007-2017. https://open.alberta.ca/dataset/e771c4d4-c677-45a2-9bff-7424f71d0a35/resource/e761dd24-275a-44ec-a51d-75b8190305c8/download/immunization-strategy-07.pdf. Accessed April 20, 2018.

[CR13] Government of Alberta. (2022). Population. https://economicdashboard.alberta.ca/Population. Accessed March 21, 2022.

[CR14] Gust DA, Darling N, Kennedy A, Schwartz B (2008). Parents with doubts about vaccines: Which vaccines and reasons why. Pediatrics.

[CR15] Hermann JS, Simmonds KA, Bell CA, Rafferty E, MacDonald SE (2019). Vaccine coverage of children in care of the child welfare system. Canadian Journal of Public Health.

[CR16] Hill HA, Elam-Evans LD, Yankey D, Singleton JA, Kolasa M (2015). National, state, and selected local area vaccination coverage among children 19-35 months-United States, 2014. Morbidity and Mortality Weekly Report.

[CR17] Kershaw T, Suttorp V, Simmonds K, St Jean T (2014). Outbreak of measles in a non-immunizing population, Alberta 2013. Canadian Communicable Disease Report.

[CR18] Kulig JC, Meyer CJ, Hill SA, Handley CE, Lichtenberger SM, Myck SL (2002). Refusal and delay of immunization within Southwest Alberta. Understanding alternative beliefs and religious perspectives. Canadian Journal of Public Health.

[CR19] Lee C, Robinson JL (2016). Systematic review of the effect of immunization mandates on uptake of routine childhood immunizations. Journal of Infection.

[CR20] Luman ET, McCauley MM, Shefer A, Chu SY (2003). Maternal characteristics associated with vaccination of young children. Pediatrics.

[CR21] MacDonald SE, Russell ML, Liu XC (2019). Are we speaking the same language? An argument for the consistent use of terminology and definitions for childhood vaccination indicators. Human Vaccines & Immunotherapeutics.

[CR22] MacDonald SE, Schopflocher DP, Vaudry W (2014). Parental concern about vaccine safety in Canadian children partially immunized at age 2: A multivariable model including system level factors. Human Vaccines & Immunotherapeutics.

[CR23] Matkin, A., Simmonds, K., & Suttorp, V. (2014). Measles-containing vaccination rates in Southern Alberta. *The Public Health Agency of Canada, CCDR, 40*(12). 10.14745/ccdr.v40i12a03 10.14745/ccdr.v40i12a03 PMC5864432 29769846

[CR24] McKee C, Bohannon K (2016). Exploring the reasons behind parental refusal of vaccines. The Journal of Pediatric Pharmacology and Therapeutics.

[CR25] Nestander M, Dintaman J, Susi A, Gorman G, Hisle-Gorman E (2018). Immunization completion in infants born at low birth weight. Journal of the Pediatric Infectious Diseases Society.

[CR26] Niederhauser VP, Stark M (2005). Narrowing the gap in childhood immunization disparities. Pediatric Nursing.

[CR27] Pearce A, Elliman D, Bedford H, Law C (2008). Residential mobility and uptake of childhood immunisations: Findings from the UK Millennium Cohort Study. Vaccine.

[CR28] Rafferty, E., Guo, X., McDonald, B., Svenson, L. W., & MacDonald, S. E. (2019). Measurement of coverage, compliance and determinants of uptake in a publicly funded rotavirus vaccination programme: A retrospective cohort study. *BMJ Open, 9*(11). 10.1136/bmjopen-2019-031718 10.1136/bmjopen-2019-031718 PMC6830662 31678951

[CR29] Rehn M, Uhnoo I, Kühlmann-Berenzon S, Wallensten A, Sparén P, Netterlid E (2016). Highest vaccine uptake after school-based delivery- A country level evaluation of the implementation strategies for HPV catch-up vaccination in Sweden. PLoS ONE.

[CR30] Riddell MA, Leydon JA, Ugoni A, Kelly HA (2001). A serosurvey evaluation of the school-based measles ‘catch-up’ immunization campaign in Victorian school-aged children. Australian and New Zealand Journal of Public Health.

[CR31] Sahni, V., Lai, F. Y., & MacDonald, S. E. (2014). Neonatal vitamin K refusal and non-immunization. *Pediatrics, 134*(3). 10.1542/peds.2014-1092 10.1542/peds.2014-1092 25136042

[CR32] Saini V, MacDonald SE, McNeil DA, MacDonald SW, Kellner JD, Edwards SA, Stagg V, Tough S (2017). Timeliness and completeness of routine childhood vaccinations in children by two years of age in Alberta, Canada. Canadian Journal of Public Health.

[CR33] Smith PJ, Chu SY, Barker LE (2004). Children who have received no vaccines: Who are they and where do they live?. Pediatrics.

[CR34] Van Pelt, D. (2015). Home schooling in Canada: The current picture – 2015 edition. https://www.fraserinstitute.org/sites/default/files/home-schooling-in-canada-2015-rev2.pdf. Accessed May 6, 2022.

[CR35] Waye A, Jacobs P, Tan B (2013). The impact of the universal infant varicella immunization strategy on Canadian varicella-related hospitalization rates. Vaccine.

[CR36] Williams N, Woodward H, Majeed A, Saxena S (2011). Primary care strategies to improve childhood immunisation uptake in developed countries: Systematic review. Journal of the Royal Society of Medicine Short Reports.

[CR37] Wilson SE, Chung H, Schwartz KL, Guttmann A, Deeks SL, Kwong JC, Crowcroft NS, Wing L, Tu K (2018). Rotavirus vaccine coverage and factors associated with uptake using linked data: Ontario, Canada. PLoS One.

