# Correction: Immunization status of children at kindergarten entry in Alberta, Canada

**DOI:** 10.17269/s41997-022-00679-9

**Published:** 2022-08-19

**Authors:** Manisha Dhungana, Matthias Hoben, Celine O’Brien, Shannon E. MacDonald

**Affiliations:** 1 Faculty of Nursing, Edmonton Clinic Health Academy, University of Alberta, 11405-87 Ave NW, Edmonton, Alberta T6G 1C9 Canada; 2 Immunization & Communicable Disease Control, Alberta Health, Edmonton, Alberta Canada

**Correction: Canadian Journal of Public Health**

10.17269/s41997-022-00663-3

This article was updated to correct the copyright holder.

